# SNP rs12982687 affects binding capacity of lncRNA UCA1 with miR-873-5p: involvement in smoking-triggered colorectal cancer progression

**DOI:** 10.1186/s12964-020-0518-0

**Published:** 2020-03-06

**Authors:** Yang Fu, Yizheng Zhang, Jinyuan Cui, Ge Yang, Sanfei Peng, Wunan Mi, Xiangya Yin, Yang Yu, Jianwu Jiang, Qi Liu, Yiyu Qin, Wen Xu

**Affiliations:** 1 Department of Gastrointestinal Surgery, The First Affiliated Hospital of Zhengzhou University, No. 1 Jianshe East Road, Zhengzhou City, 450052 Henan Province China; 2 Department of Ophthalmology, The First Affiliated Hospital of Zhengzhou University, Zhengzhou, 450052 Henan China; 3 Research Centre of Biomedical Technology, Jiangsu Vocational College of Medicine, No. 283 Jianfang South Road, Yancheng City, Jiangsu Province, 224000 China; 4 State Key Laboratory of Bioreactor Engineering & Shanghai Key Laboratory of New Drug Design, School of Pharmacy, East China University of Science and Technology, No. 130 Meilong Road, Shanghai, 200237 China

**Keywords:** CRC, lncRNA UCA1, Single nucleotide polymorphism, Gene-environment interaction, miR-873-5p, Nicotine, Cell proliferation, Cell migration

## Abstract

**Background:**

This investigation was arranged to elucidate whether single nucleotide polymorphisms (SNPs) of lncRNA UCA1 was implicated in elevating colorectal cancer (CRC) risk by interacting with environmental exposures.

**Methods:**

LncRNASNP database was firstly adopted to predict SNPs that possibly affected binding of UCA1 with miRNAs and then the interactive effect of SNPs and environmental exposure on CRC risk was evaluated by recurring to type 2 gene-environment interactions (GEI) model. Besides, MTT assay, colony formation assay, transwell assay and wound healing assay were performed to assess the activity of CRC cell lines which carried distinct genotypes of specific SNPs. The impact of nicotine on activity of CRC cells was also appraised.

**Results:**

SNP rs12982687 of UCA1 intervened in the binding capacity of UCA1 with several miRNAs, especially miR-873-5p. MiRNAs regulated by UCA1, as predicted by mirPath software, shared genes that were enriched in HIF1 signaling pathway. Moreover, homozygote TT of rs12982687 reduced CRC risk among smokers, and CRC cells that carried rs12982687 (CC) displayed strong migration and invasion. By contrast, miR-873-5p mimic, which reduced UCA1 expression, delayed metastasis of CRC cells (all *P* < 0.05). Additionally, nicotine not merely elevated UCA1 and HIF-1α expressions in CRC cells, but also facilitated proliferation and metastasis of CRC cells (*P* < 0.05).

**Conclusions:**

SNP rs12982687 was involved in smoking-triggered CRC progression, given its influence on UCA1's binding with miR-873-5p and HIF-1 signaling.

## Background

Colorectal cancer (CRC), a malignancy deriving from epithelium of colonic mucosa, was a multi-factorial disorder triggered by environmental and genetic hazards [1]. Annually 8% of all newly-diagnosed tumor cases in USA were identified as CRC, and CRC stood out as the 3rd leading account for cancer-induced death [2, 3]. Delays in diagnosis might explain this unsatisfactory prognosis, which was ascribed to that symptoms of early-stage CRC were tough to perceive [4]. Hence, it seemed extremely significant to screen particularized CRC biomarkers, which might help to elucidate CRC pathogenesis and figure out effective strategies for CRC diagnosis and treatment.

In addition to possessing protein-binding sites, lncRNAs could also dynamically and specifically acted upon DNAs and RNAs based on their sophisticated secondary structure [5], which altogether caused expressional change of pathogenic genes and ultimately disease progression [6]. Of note, there were huge potential for lncRNAs, despite their inability to encode proteins, to boost or decelerate neoplastic development (e.g. CRC) [7–10]. For instance, highly-expressed lncRNA CRNDE was an indicator of advanced CRC, and knockout of lncRNA CRNDE could induce in-vivo and in-vitro apoptosis of CRC cells [11, 12]. Moreover, incremental expression of CCAT1 and CCAT2 might enable aggressive growth and intrusion of CRC cells [13, 14], and lncRNA MIR100HG was able to reinforce cetuximab-tolerance of CRC cells via activation of Wnt/β-catenin signaling [15]. With regard to lncRNA urothelial carcinoma associated 1 (UCA1), its high expression could reflect poor survival of CRC patients, and artificially raising its intracellular expression might powerfully enhance epithelial-mesenchymal transition (EMT) and radio-resistance of CRC cells [16, 17]. Apart from CRC, the oncogenic function of UCA1 was also embodied in non-small cell lung cancer [18], bladder cancer [19], breast cancer [20], tongue squamous cell carcinoma [21] and esophageal cancer [22], a portion of which were achieved through its interplay with specific miRNAs.

There were several regulatory manners between lncRNAs and miRNAs [23, 24]. An emerging theory proposed that single nucleotide polymorphisms (SNPs) were competent in altering the secondary structure of lncRNAs [25], which produced sizeable effects on the interaction of lncRNA with corresponding miRNAs and ultimately heightened cancer risk. For instance, SNP rs11752942 (A > G) was estimated to interfere with lincRNA-uc003opf.1 expression through binding with miR-149, which altogether facilitated progression of esophageal squamous cell carcinoma [26]. This rule might also be applicable to lncRNA UCA1, which suggested that certain SNPs of UCA1 counted in regulating cancer (e.g. CRC) risk by interfering with binding of UCA1 to miRNAs [25]. Besides the genetic factors, environmental exposures were also estimated to enlarge cancer risk [27, 28]. And there was a speculation that SNPs could interact with environmental exposures to multiply disease risk [29–33]. It was thus hypothesized that risky SNPSs in UCA1 might intensify risk via its interaction with environmental hazards, although relevant molecular explanations remained far from complete.

Hence, this investigation was aimed at locating SNPs that might disturb the binding capacity of UCA1 to CRC-regulating miRNAs. Besides, environmental parameters that aggravated or relieved genetic impacts on CRC risk were also explored, which might conduce to developing effective strategies for diagnosing and treating CRC.

## Materials and methods

### Collection of clinical samples

From April 2012 to July 2013, 762 patients pathologically examined as CRC were recruited from the First Affiliated Hospital of Zhengzhou University. Their severity was classified according to the TNM staging system revised by Union for International Cancer Control (UICC). Both cancer tissues and corresponding normal mucosa (≥10 cm distant from tumor tissues) were excised from the patients during surgery. The included CRC patients all met following requirements: 1) their age ranged from 18 years old to 75 years old; 2) they received none of chemotherapy, targeted therapy or radiotherapy pre-operatively; 3) their follow-up information was complete; and 4) their RNA samples were qualified. Correspondingly, those diagnosed with following diseases would be excluded from this project, including familial adenomatous polyps, other colonic disorders (e.g. ulcerative colitis, colon tuberculosis and Crohn’s disease), other primary cancers and immune-deficient disorders (e.g. HIV and AIDS). For another, 1408 healthy Chinese who took medical examinations in the First Affiliated Hospital of Zhengzhou University were included into the control group, and they were without any history of tumors, liver/kidney dysfunction or any other smoking-induced disorders. Last but not the least, this study was approved by the First Affiliated Hospital of Zhengzhou University and its ethics committee, and all the participants have signed informed consents.

### Follow-up of CRC patients

The CRC patients were followed up for 4 years through household survey, questionnaire-assisted investigation and telephone. Forty-one CRC patients quitted midway from this project, owing to loss of contact. The follow-up period started from the time point when diagnosis was confirmed until death of the patient. The survival time of CRC patients ranged from 0.34 month to 50 months, with a median value of 34.29 months.

### Selection of SNPs

We screened SNPs of UCAl mainly following four procedures. In the first place, UCAl was positioned through retrieving UCSC human GRCh37hg19 database (http://genome.ucsc.edu). Secondly, SNPs within UCAl were filtrated from database of 1000 Genomes Project, and ones that accorded with following three conditions were selected: 1) their genetic typing rate was > 95%; b) their minor allele frequency (MAF) was > 0.05; and c) their Hardy-Weinberg equilibrium (HWE) was > l × 10^− 5^. Thirdly, SNPs situated in the intron region of UCAl, which were marked based on NCBI dbSNP database (https//www.ncbi.nlm.nih.gov/projects/SNP), were removed. Finally, SNPs that might affect binding of UCA1 with miRNAs were screened, assisted by lncRNASNP online database (http://bioinfo.1ife.hust.edu.cn/lncRNASNP).

### Genotyping of SNPs

Around 5 ml venous blood was taken from each subject in the morning and genomic DNAs were extracted from blood utilizing blood-genomic DNA extraction kit (Tiangen, China). The DNAs were then genotyped on the TaqMan platform (ABI 7900HT Real-time PCR System, Applied Biosystems), and Genepharma (China) was entrusted to design primers of rs11085996 (F: 5′-GGCTTTTGTAAACAGAGGCGTTT-3′; R: 5′-CACAGAGCAGTGGCTGACTCTT-3′) and rs12982687 (F: 5′-CAGGAGCCAAGAAGTCTGGAG-3′; R: 5′-GGAACAAGTCATAATGGTGGACAAG-3′).

### Cell culture

A couple of human CRC cell lines (i.e. SW480 and SW620) were purchased from the Chinese Academy of Sciences (Shanghai, China). They were cultured in 5% CO_2_ and 95% humidity at 37 °C, and the culture solution was managed as RPMI 1640 medium (Gibo, USA), which was inclusive of 10% fetal bovine serum (FBS), 100 U/ml penicillin and 100 μg/ml streptomycin. Until cells grew to 90% confluence, they were prepared for following experiments.

### Cell transfection

CRC cells at the density of 2 × 10^5^ per well were inoculated within 2 ml DMEM complete medium. When cell density achieved 70–80%, pcDNA3.1-UCA1, si-UCA1, si-NC, miR-873-5p mimic and miR-873-5p inhibitor (Ribobio, China) were separately transfected into each well, according to instructions particularized by Lipofectamine 2000 kit (Invitrogen, USA).

### Luciferase reporter gene assay

The UCA1 fragments that separately carried allele T and allele C, synthesized by Generay Biotech (Shanghai, China), were firstly digested by Xho*I*/Not*I*, and were then ligated to the psiCHECK-2 vector. Then the two vectors were, respectively, co-transfected with miR-873-5p mimic, miR-1207-5p mimic and miR-584 mimic (Ribobio, China) into CRC cells. Twenty-four hours later, luciferase activity of the cells was examined, as per the guidance of dual reporter gene detection kit (Promega, USA).

### Quantitative reverse transcription-polymerase chain reaction (qRT-PCR)

Aided by TRIzol (Invitrogen, USA), total RNAs were extracted from CRC tissues and cells. Then the gathered RNAs were reversely transcribed into cDNAs through usage of First Strand cDNA Synthesis kit (TaKaRa, Japan), and the cDNAs of UCA1 were amplified as per specifications of SYBR Premix Ex Taq II kit (TaKaRa, Japan). Regarding miR-1207-5p, miR-873-5p and miR-584, the All-in-One™ miRNA qRT-PCR detection kit (GeneCopoeia, USA) was employed to obtain their amplified cDNAs. With primers shown in Additional file 2: Table S1, UCA1, miR-1207-5p, miR-873-5p and miR-584 levels were monitored on the real-time fluorescence qPCR instrument (model: LightCycle®96, Roche, USA), and their relative expression was calculated on the basis of 2^-ΔΔCt^ method. Besides, GAPDH and U6 were, respectively, set as the internal reference for UCA1 and miRNAs (i.e. miR-1207-5p, miR-873-5p and miR-584).

### Western blotting

The CRC cells, having been washed with pre-cooled PBS for twice, were dissociated by protease inhibitor-containing RIPA lysate. Then the concentration of extracted total protein was examined by referring to bicinchoninic acid (BCA) method. Exactly 25 μg protein of each sample was taken to carry out 10% sodium dodecyl sulfate-polyacrylamide gel electrophoresis (SDS-PAGE), after which the samples were transferred onto the polyvinylidene fluoride (PVDF) membrane. After 2-h blockage of the samples with 5% skimmed milk powder, mouse anti-human primary antibodies against E-cadherin, N-cadherin and Vimentin (1:1000, Abcam, USA) were supplemented to incubate samples at 4 °C for overnight. With TBST applied to rinse proteins for 3 times, goat anti-mouse secondary antibodies labeled by horseradish peroxidase (HRP) (1:1000, Abcam, USA) were arranged to incubate proteins for another 1 h. Finally, development was accomplished via adoption of electrochemical luminescence, and gray values of the protein bands were analyzed by employing Image J software.

### MTT assay

Precisely 3 × 10^3^ cells of each treatment were, respectively, incubated for 0 h, 24 h, 48 h and 72 h, and then 50 μl MTT (5 g/L, Sigma, German) was prepared to treat the cells. After 4-h incubation at 37 °C, the reactions were terminated by addition of 200 μl DMSO. At last, the absorbance (A) value of each sample was measured on the ELISA-Reader (Bio-Rad, USA) at the wavelength of 490 nm.

### Colony formation assay

Exactly 3 × 10^3^ cells per well were cultured in 5% CO_2_ at 37 °C for 9 days After being fixated with 95% methanol for 10 min, the cells were stained with 0.1% methylene blue. Finally, colonies that included > 50 cells were recorded under the microscope.

### Cell apoptosis assay

Exactly 1 × 10^5^ cells of each group were re-suspended by addition of 500 μl binding buffer, in strict accordance with the guidance of Annexin V-FITC/PI double-staining kit (KeyGen Biotech, NanJing, China). Then 5 μl Annexin V-fluorescein isothiocyanate (FITC) and 5 μl propidium iodide (PI) were successively added to incubate cells. After incubation in the dark for 15 min, flow cytometry (model: FACS Calibur, BD, USA) was operated to determine apoptotic rate of the cells.

### Transwell invasion assay

The polycarbonate membrane of transwell chambers was coated with a layer of Matrigel at 37 °C for 45 min. Then CRC cells adjusted to a concentration of 4 × 10^5^/ml were added to the upper layer of Transwell chamber (Corning, USA), while 700 μl 10% FBS-containing medium was supplemented to the lower chamber. After incubating the cells in 5% CO_2_ for 24 h, the cells in the lower chamber were fixated by pre-cooled 4% paraformaldehyde for 30 min and then stained by Giemsa for 20 min. A total of 8 views were randomly selected to calculate the average cell number.

### Wound healing assay

When CRC cells inoculated into 6-well plates grew to nearly 80% confluence, a line was drawn on the back of cell plates utilizing the tip of a microsyringe (model: 10 μl). After on-going cultivation in 5% CO_2_ for 24 h, the migratory distance of the cells was analyzed utilizing Image Pro-Plus 6.0 software (Media Cybernetics, USA).

### Statistical analyses

SPSS software ver. 18 (Chicago, USA) was employed to perform all statistical analyses. The quantitative data were analyzed by virtue of independent two-tailed t test, and chi-square test was applied to compare categorical data. Logistic regression was implemented to evaluate the association of SNPs with CRC risk and prognosis. And Kaplan-Meier curves were devised to mirror survival of CRC patients, with log-rank test adopted to estimate between-group distinctions. Additionally, type 2 gene-environment interaction (GEI) model [34, 35] and multifactor dimensionality reduction (MDR) model [36] were established, so as to assess the interactive contribution of SNPs in UCA1 and environmental factors to CRC risk. Statistical significance was identified in case of *P* < 0.05.

## Results

### Screening of SNPs that affected the secondary structure of lncRNA UCA1

**Fig. 1:**
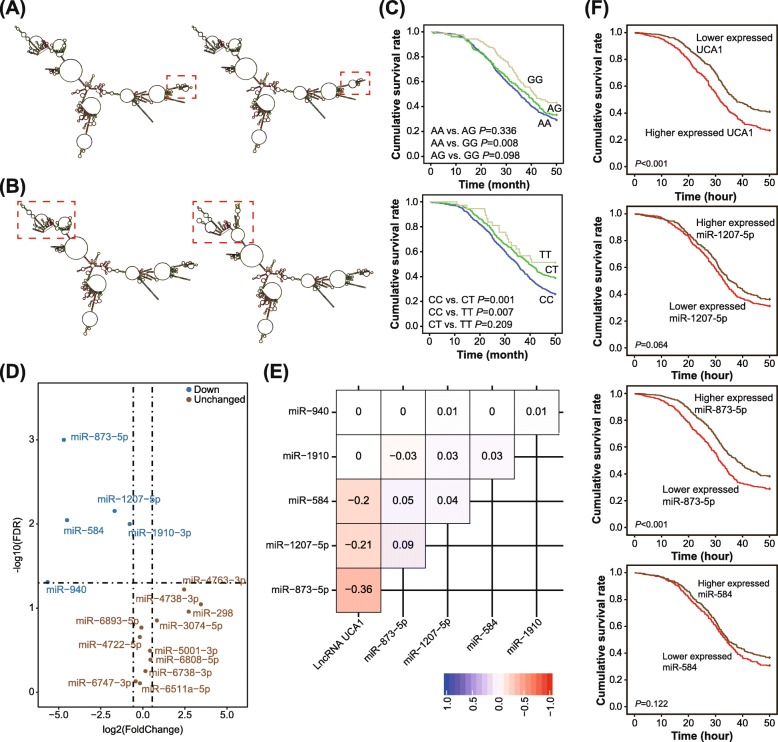
Association of single nucleotide polymorphisms (SNPs) in lncRNA UCA1 with survival of colorectal cancer (CRC) patients. **a** The secondary structure of UCA1 varied between allele C (left) and allele T (right) of rs12982687. The red block was indicative of changes in the secondary structure of UCA1. **b** The secondary structure of UCA1 differed between allele A (left) and allele G (right) of rs11085996. The red block was indicative of changes in the secondary structure of UCA1. **c** Four-year survival was compared among CRC patients who carried distinct genotypes of rs11085996 (up) and rs12982687 (down). **d** MiRNAs that possibly bound to UCA1 were quantified within CRC tissues and adjacent non-tumor tissues. The miRNAs were deemed as down-regulated when their -log10 (false discovery rate) was > 1.2 and log2 (fold change) was <− 0.5. **e** Correlations of UCA1 expression with miRNA levels were displayed by establishing correlation matrix. **f** Survival curves were depicted to assess the association of UCA1, miR-873-5p, miR-1207-5p and miR-584 expressions with CRC prognosis

According to the retrieval results from UCSC human GRCh37/hg19 database, UCA1, with a length of 7375 bp, was located within the segment of chr19:15,939,757-15,947,131. Through usage of lncRNASNP database, a couple of SNPs (i.e. rs11085996 and rs12982687) were expected to affect binding of UCA1 to certain miRNAs. Subsequently, RNAfold software was employed to single out SNPs whose mutation was capable of altering the secondary structure of UCA1. It was indicated that the secondary structure of UCA1 differed when rs12982687 (allele C vs. allele T, Fig. 1a) or rs11085996 (allele G vs. allele A, Fig. 1b) carried distinct alleles.

### Association of significant SNPs in UCA1 with CRC risk and prognosis

The CRC patients and healthy controls were well matched in terms of mean age (*P* = 0.140) and sex ratio (*P* = 0.069) (Table 1). Nevertheless, respondents characterized by smoking/alcohol consumption, low intake of fruit/vegetable and uptake of hot-temperature food/beverage were associated with incremental susceptibility to CRC, as compared with those without such habits (all *P* < 0.05). Furthermore, homozygote GG of rs11085996 appeared as a protector against CRC in comparison to genotype AA/AG (OR = 0.84, 95% CI: 0.72–0.98, *P* = 0.026), yet barely any significant association was discoverable under the allelic or dominant model (both *P* > 0.05). Regarding rs12982687, its allele T seemed capable of reducing CRC risk as relative to allele C (OR = 0.78, 95% CI: 0.67–0.90, *P* = 0.001), and carriers of genotypes TC/TT were less susceptible to CRC than those carrying homozygote CC (OR = 0.78, 95% CI: 0.62–0.97, *P* = 0.025) (Table 2). For another, CRC patients in possession of genotypes TT/CT of rs12982687 enjoyed more favorable prognosis than those with homozygote CC (*P* < 0.05), and homozygote GG of rs11085996 was associated with longer lifespan than homozygote AA (*P* = 0.008) (Fig. 1c). Considering an outstanding association of rs12982687 with CRC risk, this SNP was managed for later cellular experiments.

**Table 1:** Comparison of basic clinical features between colorectal cancer patients and healthy controls

Clinical feature	Case		Control		χ^2^	*P* value
	Number	Percentage	Number	Percentage		
Age (years)						
≥ 50	423	55.51%	735	52.20%		
<50	339	44.49%	673	47.80%	2.18	0.140
Gender						
Female	189	24.80%	301	21.38%		
Male	573	75.20%	1107	78.62%	3.32	0.069
Pathological classification						
Poor differentiation	216	28.35%	–			
Moderate differentiation	305	40.03%				
Well differentiation	241	31.63%				
TNM classification						
I	32	4.20%	–			
II	371	48.69%				
III	268	35.17%				
IV	91	11.94%				
Smoking						
Yes	592	77.69%	849	60.30%		
No	170	22.31%	559	39.70%	67.04	**< 0.001**
Alcohol						
Yes	563	73.88%	778	55.26%		
No	199	26.12%	630	44.74%	72.68	**< 0.001**
Fruit intake, > 1 serving/day						
Yes	329	43.18%	821	58.31%		
No	433	56.82%	587	41.69%	45.46	**< 0.001**
Vegetable intake, > 1 serving/day						
Yes	481	63.12%	990	70.31%		
No	281	36.88%	418	29.69%	11.70	**< 0.001**
Foods or beverages temperature						
Hot	494	64.83%	784	55.68%		
Warm	268	35.17%	624	44.32%	17.09	**< 0.001**
Smoked or pickled foods intake						
Yes	478	62.73%	834	59.23%		
No	284	37.27%	574	40.77%	2.53	0.112

The bold results indicated there is a statistically significance when its p value < 0.05.

**Table 2:** Association of single nucleotide polymorphisms within UCA1 with the risk of colorectal cancer

Gene	rs number	Allele Change	Mode	Genotype	CRC	Control	*P* value	OR (95% CI)^*^
LncRNA UCA1	rs11085996	A > G	Allelic model	A	1195	2193		1
				G	329	623	0.684	0.97 (0.83–1.13)
			Dominant model	AA	521	945		1
				AG + GG	241	463	0.213	0.88 (0.71–1.08)^a^
			Recessive model	AA+AG	674	1248		1
				GG	88	160	**0.026**	**0.84 (0.72–0.98)**^**a**^
	rs12982687	C > T	Allelic model	C	1188	2065		1
				T	336	751	**0.001**	**0.78 (0.67–0.90)**
			Dominant model	CC	463	751		1
				CT + TT	299	657	**0.025**	**0.78 (0.62–0.97)**^**a**^
			Recessive model	CC + CT	725	1314		1
				TT	37	94	0.917	0.98 (0.64–1.50) ^a^

*OR (Allelic model) = Mutant allele/Wild allele, OR (Dominant model) = (Heterozygote+Mutant homozygote)/Wild homozygote, OR (Recessive model) = Mutant homozygote/(Wild homozygote+Heterozygote), ^a^ Gender, smoking, alcohol, fruit intake, vegetable intake, food/beverage temperature and smoked food adjusted OR. The bold results indicated there is a statistically significance when its p value <0.05

### Stratified analyses regarding association of candidate SNPs in UCA1 with susceptibility to CRC *(*See Additional file 6*)*

#### Evaluating the interactive contribution of candidate SNPs in UCA1 and smoking to CRC risk by establishing GEI and MDR models *(*See Additional file 6*)*

The results of GEI model demonstrated that homozygote CC of rs12982687 could amplify the promoting effect of advanced age (≥ 50 years old) (γ = 2.80), smoking (γ = 1.98) and up-take of hot-temperature food/beverage (γ = 2.75) on CRC susceptibility (Table 3). With respect to rs11085996, smoking (γ > 1) and hot-temperature food intake (γ > 1) both made AG/AA carriers more readily plagued by CRC, yet intake of fruit largely reduced the risk of developing CRC (γ = − 81.58) (Table 4).

**Table 3:** Interactive analysis between rs12982687 within UCA1 and living habits on susceptibility to colorectal cancer

Clinical feature	Genotype	Characteristics	Case	Control	*P* value	OR (95% CI)	βe	βeg	γ value^*^
Age (years)	TT	<50	32	79			0.00		
	CT	<50	42	86	0.506	1.21 (0.69–2.09)	0.19		
	CC	<50	265	508	0.255	1.29 (0.83–1.99)	0.25		
	TT	≥50	5	15	0.726	0.82 (0.28–2.45)	0.00	−0.19	–
	CT	≥50	220	477	0.563	1.14 (0.73–1.77)	0.19	0.13	0.68
	CC	≥50	198	243	**0.002**	**2.01 (1.28–3.16)**	0.25	0.70	2.80
Gender	TT	Female	13	40			0.00		
	CT	Female	65	110	0.090	1.82 (0.91–3.65)	0.60		
	CC	Female	111	151	**0.015**	**2.26 (1.16–4.43)**	0.82		
	TT	Male	24	54	0.436	1.37 (0.62–3.01)	0.00	0.31	–
	CT	Male	197	453	0.377	1.34 (0.70–2.56)	0.60	0.29	0.49
	CC	Male	352	600	0.067	1.81 (0.95–3.42)	0.82	0.59	0.72
Smoking	TT	None	8	39			0.00		
	CT	None	43	185	0.768	1.13 (0.49, 2.60)	0.12		
	CC	None	146	358	0.081	1.99 (0.91, 4.36)	0.69		
	TT	Often	29	55	**0.033**	**2.57 (1.06, 6.22)**	0.00	0.94	–
	CT	Often	219	378	**0.007**	**2.82 (1.30, 6.15)**	0.12	1.04	8.65
	CC	Often	317	393	**< 0.001**	**3.93 (1.81, 8.54)**	0.69	1.37	1.98
Alcohol	TT	None	25	18			0.00		
	CT	None	81	332	**< 0.001**	**0.18 (0.09–0.34)**	−1.74		
	CC	None	99	298	**< 0.001**	**0.24 (0.13–0.46)**	−1.43		
	TT	Often	12	76	**< 0.001**	**0.11 (0.05–0.27)**	0.00	−2.17	–
	CT	Often	181	231	0.075	0.56 (0.30–1.07)	−1.74	−0.57	0.33
	CC	Often	364	453	0.081	0.58 (0.31–1.08)	−1.43	− 0.55	0.38
Fruit intake, > 1 serving/day	TT	Yes	8	79			0.00		
	CT	Yes	146	166	0.913	0.97 (0.61, 1.55)	− 0.03		
	CC	Yes	279	342	0.351	1.25 (0.78, 1.99)	0.22		
	TT	No	29	15	0.443	1.45 (0.56, 3.79)	0.00	0.37	–
	CT	No	116	397	**0.006**	**1.97 (1.21, 3.20)**	−0.03	0.68	−22.59
	CC	No	184	409	**0.002**	**2.05 (1.30, 3.21)**	0.22	0.72	3.26
Vegetable intake, > 1 serving/day	TT	Yes	12	92			0.00		
	CT	Yes	177	383	**< 0.001**	**4.14 (2.22, 7.74)**	1.42		
	CC	Yes	294	515	**< 0.001**	**3.93 (2.12, 7.30)**	1.37		
	TT	No	25	2	**< 0.001**	**95.83 (20.12, 456.46)**	0.00	4.56	–
	CT	No	85	180	**0.011**	**2.34 (1.20, 4.59)**	1.42	0.85	0.60
	CC	No	169	236	**< 0.001**	**6.46 (3.44, 12.14)**	1.37	1.87	1.36
Foods or beverages temperature	TT	Warm	23	61			0.00		
	CT	Warm	126	310	0.778	1.08 (0.64–1.82)	0.08		
	CC	Warm	119	253	0.410	1.25 (0.74–2.11)	0.22		
	TT	Hot	14	33	0.769	1.13 (0.51–2.47)	0.00	0.12	–
	CT	Hot	136	253	0.182	1.43 (0.85–2.41)	0.08	0.35	4.43
	CC	Hot	344	498	**0.016**	**1.83 (1.11–3.02)**	0.22	0.61	2.75
Smoked or pickled foods intake	TT	None	25	47			0.00		
	CT	None	120	277	0.448	0.81 (0.48–1.38)	−0.21		
	CC	None	150	278	0.957	1.01 (0.60–1.71)	0.01		
	TT	Often	12	47	0.069	0.48 (0.22–1.07)	0.00	−0.73	–
	CT	Often	142	286	0.797	0.93 (0.55–1.58)	− 0.21	− 0.07	0.33
	CC	Often	313	473	0.397	1.24 (0.75–2.06)	0.01	0.22	21.84

*The interaction coefficient (i.e. γ) was the result of βeg divided by βe. In case of **γ** > 1, genetic factors amplified the effect of environmental exposures; in case of γ > 1, genetic factors weakened the effect of environmental exposures; in case of **γ** = 1, no interaction was present between genetic factors and environmental exposures. When the environmental exposure was detrimental, genetic factors were deemed to be strongly protective in case of βe > 0 and γ < 0. The bold results indicated there is a statistically significance when its p value <0.05

**Table 4:** Interactive analysis between rs11085996 within UCA1 and living habits on susceptibility to colorectal cancer

Clinical feature	Genotype	Classifications	Case	Control	*P* value	OR (95% CI)	βe	βeg	γ value*
Age (years)	GG	<50	64	130			0.00		
	AG	<50	35	46	0.108	1.55 (0.91–2.63)	0.44		
	AA	<50	240	497	0.911	0.98 (0.70–1.37)	−0.02		
	GG	≥50	24	30	0.120	1.63 (0.88–3.00)	0.00	0.49	–
	AG	≥50	118	257	0.712	0.93 (0.64–1.35)	0.44	−0.07	−0.16
	AA	≥50	281	448	0.155	1.27 (0.91–1.78)	−0.02	0.24	−12.11
Gender	GG	Female	42	34			0.00		
	AG	Female	46	92	**0.002**	**0.40 (0.23–0.72)**	−0.90		
	AA	Female	101	175	**0.003**	**0.47 (0.28–0.78)**	−0.76		
	GG	Male	46	126	**< 0.001**	**0.30 (0.17–0.52)**	0.00	−1.22	–
	AG	Male	107	211	**< 0.001**	**0.41 (0.25–0.68)**	−0.90	−0.89	1.00
	AA	Male	420	770	**< 0.001**	**0.44 (0.28–0.70)**	−0.76	− 0.82	1.08
Smoking	GG	None	10	37			0.00		
	AG	None	40	186	0.564	0.80 (0.37–1.73)	−0.22		
	AA	None	147	359	0.258	1.52 (0.73–3.13)	0.42		
	GG	Often	78	123	**0.024**	**2.35 (1.10–4.99)**	0.00	0.85	–
	AG	Often	113	117	**< 0.001**	**3.57 (1.70–7.53)**	−0.22	1.27	−5.54
	AA	Often	798	586	**< 0.001**	**5.04 (2.49–10.21)**	0.42	1.62	3.85
Alcohol	GG	None	24	19			0.00		
	AG	None	45	200	**< 0.001**	**0.18 (0.09–0.35)**	−1.73		
	AA	None	136	429	**< 0.001**	**0.25 (0.13–0.47)**	−1.38		
	GG	Often	64	141	**0.002**	**0.36 (0.18–0.70)**	0.00	−1.02	–
	AG	Often	108	103	0.580	0.83 (0.43–1.61)	−1.73	−0.19	0.09
	AA	Often	385	516	0.091	0.59 (0.32–1.09)	−1.38	− 0.53	0.30
Fruit intake, > 1 serving/day	GG	Yes	36	96			0.00		
	AG	Yes	73	135	0.132	1.44 (0.89, 2.32)	0.36		
	AA	Yes	220	590	0.979	0.99 (0.66, 1.50)	−0.01		
	GG	No	52	64	**0.004**	**2.17 (1.28, 3.68)**	0.00	0.77	–
	AG	No	80	168	0.315	1.27 (0.80, 2.02)	0.36	0.24	0.66
	AA	No	301	355	**< 0.001**	**2.26 (1.50, 3.42)**	−0.01	0.82	−81.58
Vegetable intake, > 1 serving/day	GG	Yes	23	117			0.00		
	AG	Yes	147	251	**< 0.001**	**2.98 (1.82–4.87)**	1.09		
	AA	Yes	311	622	**< 0.001**	**2.54 (1.59–4.06)**	0.93		
	GG	No	65	43	**< 0.001**	**7.69 (4.26–13.87)**	0.00	2.04	–
	AG	No	6	52	0.271	0.59 (0.23–1.53)	1.09	−0.53	−0.49
	AA	No	210	323	**< 0.001**	**3.31 (2.05–5.34)**	0.93	1.20	1.29
Foods or beverages temperature	GG	Warm	18	60			0.00		
	AG	Warm	65	102	**0.015**	**2.12 (1.15–3.92)**	0.75		
	AA	Warm	185	462	0.305	1.33 (0.77–2.32)	0.29		
	GG	Hot	70	100	**0.006**	**2.33 (1.27–4.29)**	0.00	0.85	–
	AG	Hot	88	201	0.202	1.46 (0.81–2.62)	0.75	0.38	0.50
	AA	Hot	336	483	**0.002**	**2.32 (1.34–4.00)**	0.29	0.84	2.90
Smoked or pickled foods intake	GG	None	35	48			0.00		
	AG	None	31	87	**0.018**	**0.49 (0.27–0.89)**	−0.72		
	AA	None	229	467	0.092	0.67 (0.42–1.07)	−0.40		
	GG	Often	53	112	0.119	0.65 (0.38–1.12)	0.00	−0.43	–
	AG	Often	122	216	0.305	0.77 (0.48–1.26)	−0.72	− 0.26	0.35
	AA	Often	292	478	0.450	0.84 (0.53–1.33)	−0.40	−0.18	0.44

*: The interaction coefficient (i.e. γ) was the result of βeg divided by βe. In case of γ > 1, genetic factors amplified the effect of environmental exposures; in case of γ > 1, genetic factors weakened the effect of environmental exposures; in case of γ = 1, no interaction was present between genetic factors and environmental exposures. When the environmental exposure was detrimental, genetic factors were deemed to be strongly protective in case of βe > 0 and γ < 0. The bold results indicated there is a statistically significance when its p value <0.05

### Mutation of rs12982687 disordered binding of UCA1 to certain CRC-specific miRNAs

As predicted by LncRNASNP2 database, rs12982687C > T might disturb the binding capacity of UCA1 to some miRNAs (http://bioinfo.life.hust.edu.cn/lncRNASNP#!/snp_info?snp=rs12982687). Among the miRNAs, miR-873-5p, miR-1207-5p, miR-584, miR-1910-3p and miR-940 were evidently down-regulated within CRC tissues as opposed to adjacent normal tissues, with -log10(false discovery rate) > 1.2 and log2(fold change) < − 0.5 as the screening criterion (Fig. 1d). More than that, expressions of miR-873-5p (r_s_ = − 0.36), miR-1207-5p (r_s_ = − 0.21) and miR-584 (r_s_ = − 0.20) were separately negatively correlated with UCA1 expression among the recruited CRC patients, as illustrated by the correlation matrix (Fig. 1e). Nevertheless, merely UCA1 and miR-873-5p were significant biomarkers for predicting the prognosis of CRC patients (both *P* < 0.05) (Fig. 1f).

With the aid of mirPath v.3 (http://snf-515788.vm.okeanos.grnet.gr/) software, KEGG pathways enriched by miR-873-5p-, miR-1207-5p- and miR-584-targeted genes were figure out, and the top 5 pathways were revealed as viral carcinogenesis (N_gene_ = 24), proteoglycans in cancer (N_gene_ = 18), HIF-1 signaling pathway (N_gene_ = 18), adherens junction (N_gene_ = 16) and prostate cancer (N_gene_ = 15) (Additional file 5: Table S4). Notably, the HIF-1 signaling was implicated in the pathogenesis of smoking-induced disorders [37–39] and also CRC development [40]. Since that smoking might interact with rs12982687 of UCA1 to induce CRC risk (Additional file 1: Figure S1, Additional file 4: Table S3), there was a possibility that rs12982687 might influence HIF-1 signaling by disturbing binding of UCA1 to miR-873-5p, miR-1207-5p and miR-584.

### Mutation of rs12982687 altered binding capacity of UCA1 to miR-873-5p and also HIF-1α expression in CRC cells

**Fig. 2:**
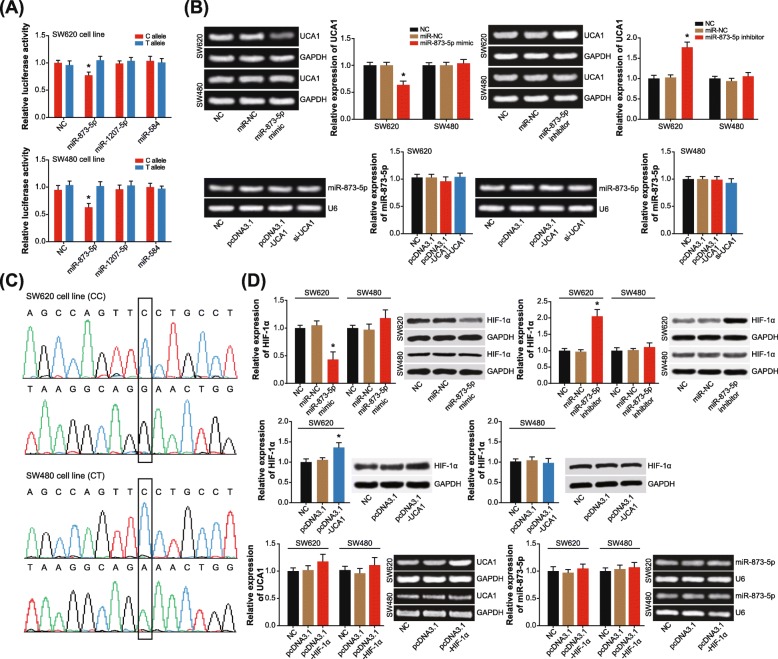
Single nucleotide polymorphism (SNP) rs12982687 (C > T) affected binding of UCA1 with miRNAs in colorectal cancer (CRC) cells. **a** The luciferase activity of CRC cells was compared between allele T + miR-873-5p/miR-1207-5p/miR-584 mimic group and allele C + miR-873-5p/miR-1207-5p/miR-584 mimic group. *: *P* < 0.05 when compared with allele T + miR-873-5p/miR-1207-5p/miR-584 mimic group. **b** UCA1 expression was determined when miR-873-5p mimic, miR-873-5p inhibitor or miR-NC was transfected into CRC cells, and miR-873-5p expression was measured among CRC cells of pcDNA3.1, pcDNA3.1-UCA1, si-UCA1 and NC groups. *: *P* < 0.05 when compared with NC group. **c** The genotype of rs12982687 (C > T) was determined within SW620 cell line and within SW480 cell line. **d** Expressional change of HIF-1α in SW620 and SW480 cell lines was determined in case of altered expression of UCA1 and miR-873-5p, and expressions of UCA1 and miR-873-5p were monitored when HIF-1α level was intentionally changed in CRC cells *: *P* < 0.05 when compared with NC group

The luciferase reporter plasmids that carried allele C/T of rs12982687 were, respectively, co-transfected with miR-873-5p mimic, miR-1207-5p mimic and miR-584 mimic into CRC cell lines (Fig. 2a). It was demonstrated that the luciferase activity of allele T + miR-873-5p mimic group was significantly distinct from that of allele C + miR-873-5p mimic group (*P* < 0.05). However, no remarkable distinction of luciferase activity was observed between allele T + miR-584 mimic group and allele C + miR-584 mimic group, as well as between allele T + miR-1207-5p mimic group and allele C + miR-1207-5p mimic group (*P* > 0.05). Therein, thus, lied a suggestion that rs12982687 C > T could diminish the binding capability of UCA1 to miR-873-5p.

In addition, UCA1 expression showed an evident reduction in miR-873-5p mimic-transfected SW620 cells, and UCA1 expression in SW620 cells was significantly promoted by transfection of miR-873-5p inhibitor (*P* < 0.05) (Fig. 2b). Nonetheless, UCA1 expression was rarely changed in SW480 cells, when miR-873-5p mimic and miR-873-5p inhibitor were transfected (*P* > 0.05). Besides, miR-873-5p expression failed to undergo any expressional change in both SW620 and SW480 cell lines, when pcDNA3.1-UCA1 and si-UCA1 were transfected (*P* > 0.05). Furthermore, the results of Sanger sequencing demonstrated that SW480 and SW620 cell lines possessed discrepant genotypes of rs12982687 (Fig. 2c), which insinuated that allele T of rs12982687 might maintain binding of UCA1 to miR-873-5p and then affected UCA1 expression.

Meanwhile, transfection of miR-873-5p mimic engendered a prominent decrease of HIF-1α expression in SW620 cell line (*P* < 0.05), and HIF-1α expression was raised after transfection of miR-873-5p inhibitor (*P* < 0.05) (Fig. 2d). Besides, transfection of pcDNA3.1-UCA1 also triggered an increase of HIF-1α level within SW620 cell line (*P* < 0.05). Interestingly, UCA1 expression was promoted by over-expressed HIF-1α in SW620 cell line (*P* > 0.05), but miR-873-5p expression seemed unaffected (*P* > 0.05). This confusing phenomenon might be owing to the feedback regulation between UCA1 and HIF-1α [41, 42], which demanded more explorations.

### Mutation of rs12982687 in UCA1 influenced EMT, proliferation and apoptosis of CRC cells

**Fig. 3:**
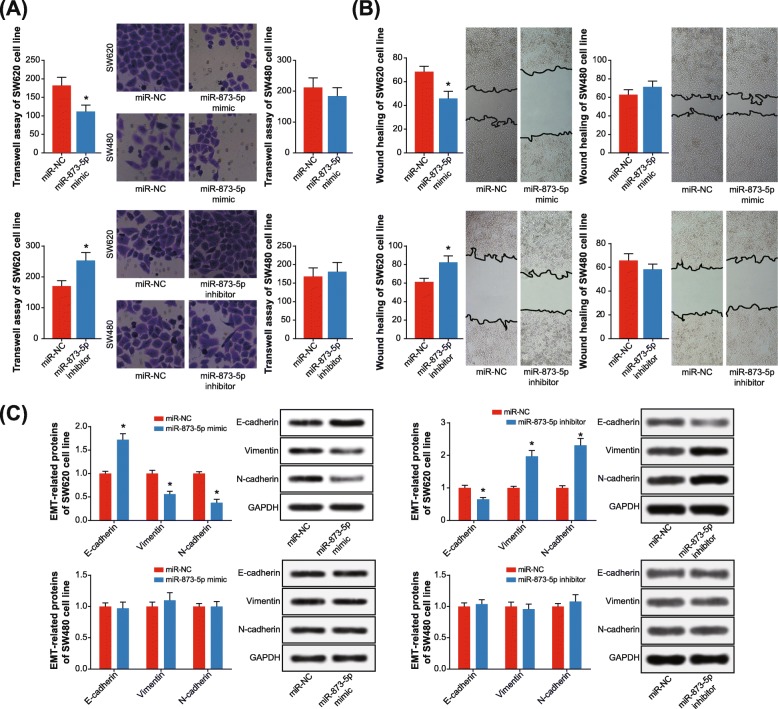
Single nucleotide polymorphism (SNP) rs12982687 (C > T) affected invasion, migration and epithelial-mesenchymal transition (EMT) of colorectal cancer (CRC) cells. **a** Invasive capability of SW620 and SW480 cell lines was assessed when miR-873-5p expression was up-regulated and down-regulated. *: *P* < 0.05 when compared with miR-NC group. **b** The migratory potency of SW620 and SW480 cell lines was compared among miR-NC, miR-873-5p mimic and miR-873-5p inhibitor groups. *: *P* < 0.05 when compared with miR-NC group. **c** Expressions of EMT-related proteins were detected within SW620 and SW480 cell lines of miR-NC, miR-873-5p mimic and miR-873-5p inhibitor groups. *: *P* < 0.05 when compared with miR-NC group

Transfection of miR-873-5p mimic was found to impair the trans-membrane potency of SW620 cell line (*P* < 0.05), rather than SW480 cell line (*P* > 0.05) (Fig. 3a). Correspondingly, SW620 cell line transfected by miR-873-5p inhibitor displayed stronger migratory potential than those transfected with plasmid vector (*P* < 0.05). Results of wound healing also presented that migration of SW620 cell line (*P* < 0.05), instead of SW480 cell line (*P* > 0.05), was weakened by miR-873-5p mimic (Fig. 3b). In stark contrast, SW620 cell line was prone to invade aggressively after being transfected by miR-873-5p inhibitor in comparison to the vector group (*P* < 0.05). What’s more, SW620 cell line (*P* < 0.05), but not SW480 cell line (*P* > 0.05), in the miR-873-5p mimic group expressed higher E-cadherin level and lower vimentin/N-cadherin level than NC group (Fig. 3c). Conversely, managing the SW620 cell line with miR-873-5p inhibitor rendered them to produce low E-cadherin level and yet high vimentin/N-cadherin level (*P* < 0.05).

**Fig. 4:**
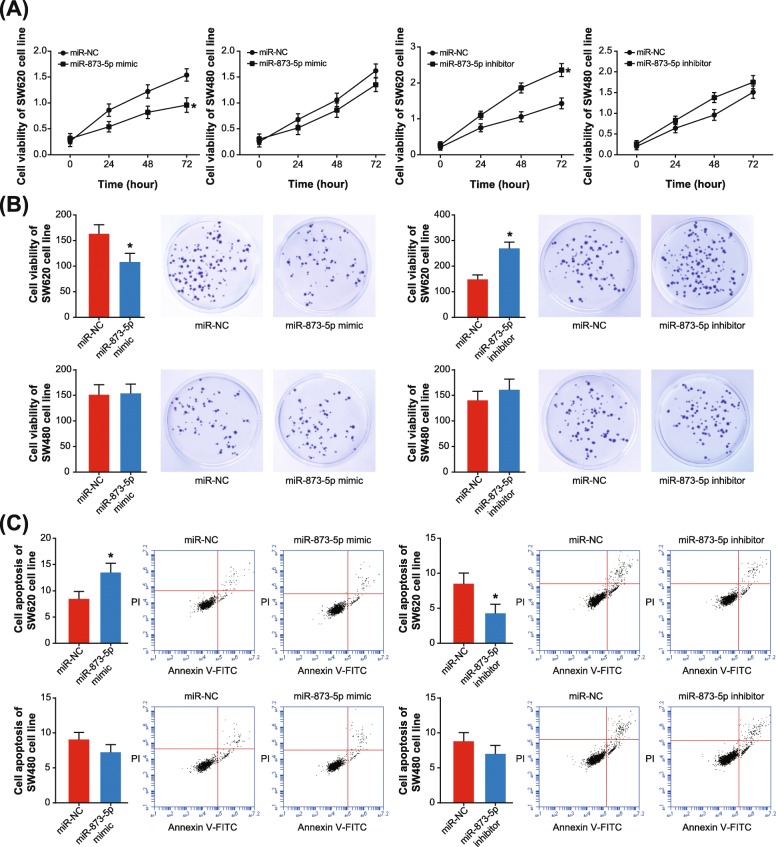
Single nucleotide polymorphism rs12982687 (C > T) affected viability, proliferation and apoptosis of colorectal cancer (CRC) cells. **a** Viability of SW620 and SW480 cell lines was measured among miR-NC, miR-873-5p mimic and miR-873-5p inhibitor groups. *: *P* < 0.05 when compared with miR-NC group. **b** Multiplication of SW620 and SW480 cell lines was contrasted among miR-NC, miR-873-5p mimic and miR-873-5p inhibitor groups. *: *P* < 0.05 when compared with miR-NC group. **c** The apoptotic rates of SW620 and SW480 cell lines were appraised among miR-NC, miR-873-5p mimic and miR-873-5p inhibitor groups. *: *P* < 0.05 when compared with miR-NC group

In addition, both proliferation and activity of SW620 cell line were hindered by miR-873-5p mimic (*P* < 0.05) (Fig. 4a-b), and the apoptotic rate of SW620 cell line was also boosted under the influence of miR-873-5p mimic (*P* < 0.05) (Fig. 4c). Treatment of miR-873-5p inhibitor, on the contrary, boosted proliferation/activity and prohibited apoptosis of SW620 cell lines (*P* < 0.05) (Fig. 4). Nonetheless, the activity of SW480 cell line was nearly unaltered, regardless of proliferation, activity or apoptosis (all *P* > 0.05). Hence, it was implied that rs12982687 might indirectly regulate UCA1 expression by affecting the binding of UCA1 to miR-873-5p, which ultimately regulated the malignant phenotype of CRC cells.

### Nicotine promoted malignancy of CRC cells and altered UCA1, miR-873 and HIF-1α expressions within CRC cells

**Fig. 5:**
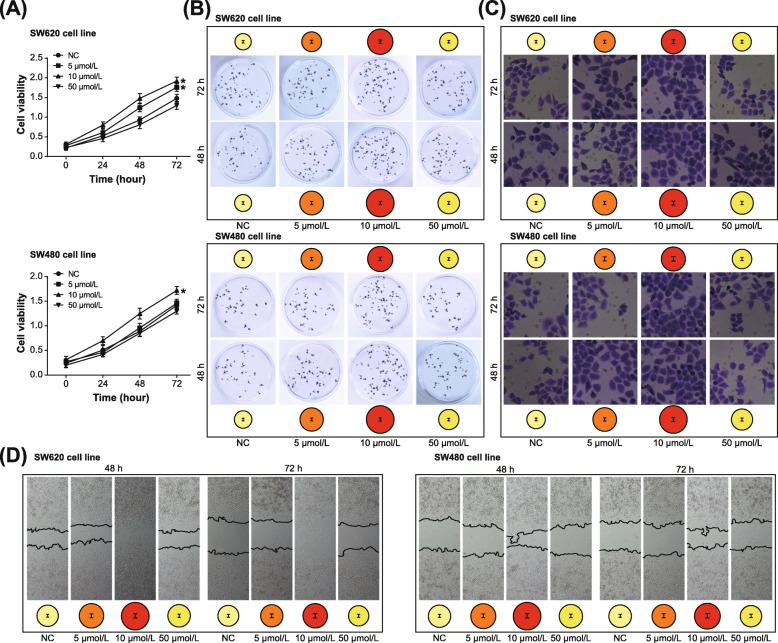
Nicotine regulated viability, proliferation, migration and invasion of colorectal cancer (CRC) cells. **a** Viability of SW620 and SW480 cell lines was assessed under treatments of 0, 5, 10 and 50 μmol/L nicotine. *: *P* < 0.05 when compared with treatment of 0 μmol/L nicotine. **b** Proliferation of SW620 and SW480 cell lines was measured under treatments of 0, 5, 10 and 50 μmol/L nicotine. *: *P* < 0.05 when compared with treatment of 0 μmol/L nicotine. **c** Invasive ability of SW620 and SW480 cell lines was evaluated under treatments of 0, 5, 10 and 50 μmol/L nicotine. *: *P* < 0.05 when compared with treatment of 0 μmol/L nicotine. **d** Nicotine at different concentrations (i.e. 0, 5, 10 and 50 μmol/L) facilitated migratory potential of SW620 and SW480 cell lines. *: *P* < 0.05 when compared with treatment of 0 μmol/L nicotine

**Fig. 6:**
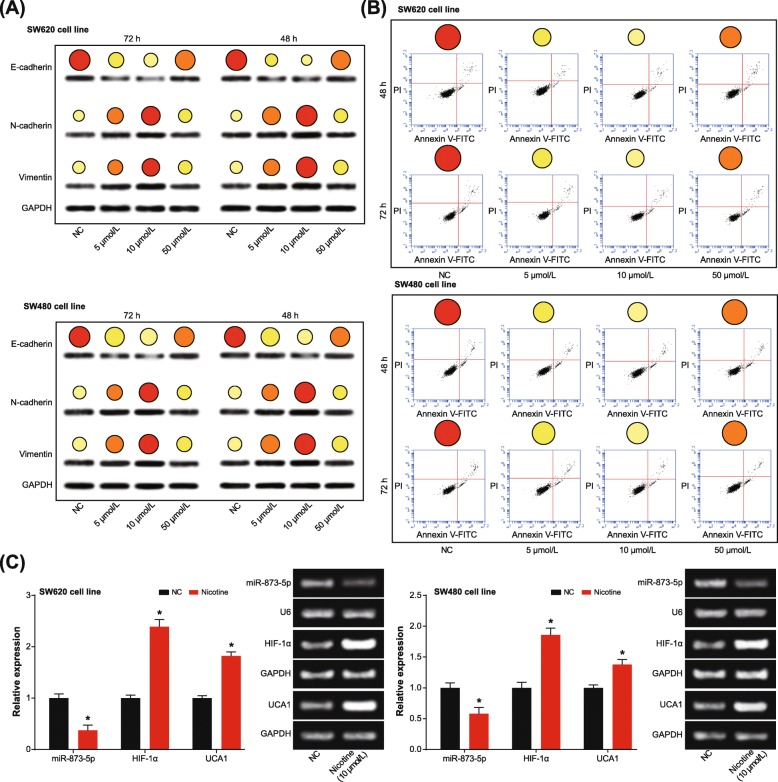
Nicotine affected epithelial-mesenchymal transition (EMT) and apoptosis of colorectal cancer (CRC) cells. **a** Expressions of EMT-specific proteins were determined under treatments of 0, 5, 10 and 50 μmol/L nicotine. *: *P* < 0.05 when compared with treatment of 0 μmol/L nicotine. **b** Nicotine at different concentrations (i.e. 0, 5, 10 and 50 μmol/L) prohibited apoptosis of SW620 and SW480 cell lines. *: *P* < 0.05 when compared with treatment of 0 μmol/L nicotine. **c** UCA1 and miR-873-5p expressions were determined under the treatment of 10 μmol/L nicotine. *: *P* < 0.05 when compared with NC group

Under treatment of 10 μmol/L nicotine, viability, migration and invasion of both SW620 and SW480 cell lines were elevated remarkably (*P* < 0.05), although the intensifying degree of SW620 cell line was more pronounced than SW480 cell line (*P* < 0.05) (Fig. 5). At the meantime, SW620 cell line was associated with lower E-cadherin expression and higher vimentin/N-cadherin expression than SW480 cell line (*P* < 0.05) (Fig. 6a). Concurrently, the apoptotic tendency of SW620 cell line was not as obvious as that of SW480 cell line (*P* < 0.05) (Fig. 6b). However, treatment of 50 μmol/L nicotine failed to significantly facilitate proliferation (Fig. 5a-b), invasion (Fig. 5c) and migration (Fig. 5d) of CRC cells, or prohibited their apoptosis (Fig. 6b) with statistical significance (*P* > 0.05). In addition, driven by 10 μmol/L nicotine, UCA1 expression was promoted, yet miR-873 expression was suppressed in both SW620 and SW480 cell lines (*P* < 0.05) (Fig. 6c). Further comparisons manifested that expressions of UCA1 and HIF-1α were altered less significantly in SW480 cell line than in SW620 cell line (*P* < 0.05).

## Discussion

CRC onset was implied as the comprehensive outcome of genetic disorders and exposure to hazardous surroundings, and here we attempted to clarify the interactive role of SNPs in UCA1 and environmental risks in enhancing CRC risk. In the first place, the GEI model was established, which disclosed that genotypes TT/CT of rs12982687 could make smokers more vulnerable to CRC risk than homozygote CC (Table 3). Then as speculated by the RNAfold software, rs12982687 (C > T) seemed capable of changing the secondary structure of UCA1 (Fig. 1a), which could result in disordered binding of UCA1 to several miRNAs (http://bioinfo.life.hust.edu.cn/lncRNASNP#!/snp_info?snp=rs12982687). Among the miRNAs, it was intriguing to discover that miR-873-5p, miR-1207-5p and miR-873-5p were pronounced biomarkers in symbolizing CRC onset (Fig. 1d), and the miR-873-5p even displayed a tight linkage with poor survival of CRC patients (Fig. 1f). There appeared a possibility that mutation of rs12982687 was responsible for altering CRC susceptibility, due to its modifying binding of UCA1 with some miRNAs, especially miR-873-5p.

To date, a broad array of evidences have corroborated the inherent association of UCA1 with etiologies of diverse neoplasms, including bladder carcinoma, breast cancer, CRC, esophageal squamous cell carcinoma, gastric cancer, hepatocellular carcinoma, melanoma, ovarian cancer and squamous-cell carcinoma [16, 21, 43–48]. Actually, UCA1 expression dwindled in adult tissues in comparison to embryonic tissues [49, 50], which, from another angle, implied the adverse effect of highly-expressed UCA1 on physiology of adults. Within this investigation, we strived to explain the CRC-promoting role of UCA1, from the perspective of its interaction with miRNAs [51]. In effect, the reciprocity of lncRNAs and miRNAs has been introduced in various disorders [25]. For instance, lncRNA ANRIL was reported to encourage proliferation of gastric carcinoma cells by silencing miR-99a and miR-449a [52], while miR-372 down-regulated expression of lncRNA HULC in hepatoma carcinoma cells by disaggregating transcriptional factors from the promoter of HULC [53]. Here it was suggested that homozygote CC of rs12982687 might prevent UCA1 expression from being suppressed by miR-873-5p (Fig. 2b), owing to which the anti-tumor role of miR-873-5p was hindered [54, 55]. Intriguingly, our study demonstrated that homozygote CC of rs12982687 could facilitate proliferation, migration and invasion of CRC cells (Figs. 3 and 4), which might be supportive of this suggestion.

Additionally, HIF-1 signaling seemed as a noteworthy pathway that mediated the role of UCA1 and miRNAs (i.e. miR-584, miR-1207-5p and miR-873-5p) in modifying CRC development, according to the results of KEGG enrichment analysis (Fig. 1d-e). There were convincing proofs that over-activation of HIF-1 could lead to aggressive proliferation and invasion of tumor cells, which was achieved via alteration of MMP9, VEGF and NDRG1 expressions [56–58]. In this investigation, we also observed that HIF-1α expression was boosted within CRC cells when UCA1 expression was up-regulated, and interestingly, a rise of UCA1 expression was detectable in case of over-expressed HIF-1α (Fig. 2d). There might be a feedback regulatory loop between UCA1 and HIF-1α, which was similar to the conclusion drawn by Li et al. [41, 42], yet mechanisms in this regard demanded in-depth exploration.

Besides the above, we proposed that smoking, which created a hypoxic micro-environment that was quite common in solid tumors [59–62], might cooperate with homozygote CC of rs12982687 to engender a super-imposed effect on CRC progression. On one hand, smoking was found to enlarge the likelihood of CRC risk among the population who carried rs12982687 (CC) (Figs. 5 and 6, Table 3). On the other hand, in-vitro experiments indicated that rs12982687 (CC) was associated with intensified proliferation, migration and invasion of CRC cells (Figs. 3 and 4). The UCA1 expression was significantly raised in SW620 and SW480 cell lines after supplementation of nicotine (Fig. 6c), although the expressional change of UCA1 was more profound in SW620 cell lines than in SW480 cell line. There was an implication that the genotype of rs12982687 was indeed correlative with the proliferative capability of CRC cells. Nonetheless, nicotine could promote proliferation of cancer cells through regulation of multifarious genetic pathways, and the expressional change of UCA1 might not reflect all carcinogenic actions of nicotine.

As a matter of fact, oxygen-poor was distinctly seen within CRC tissues [63]. For one thing, structure confusion of local tissues deformed tumor neovascularization and damaged normal blood supply of colon tissues. For another, extensive proliferation of tumor cells and expansion of tumor volume both aggravated hypoxia of CRC tissues. To survive the oxygen-deficient microenvironment, CRC cells generated a series of adaptive changes, such as variation of genomes [64]. Notably, HIF-1 signaling was among the genetic changes that happened during CRC exacerbation [65], and activation of HIF-1 signaling could powerfully enhance metastasis and drug resistance of CRC cells [66–68]. Moreover, HIF-1 signaling was also involved in the etiology of smoking-triggered cancer. In particular, knockdown of HIF-1α could interdict oncogenesis triggered by 4-(methylnitrosamino)-1-(3-pyridyl)-1-butanone (NNK), a specific component of tobacco [37]. These evidences might, from another aspect, explain the interactive effect of UCA1 and smoking on CRC risk, owing to their sharing HIF-1 signaling.

## Conclusion

SNP rs12982687 of UCA1 might influence the binding capacity of UCA1 with miR-873 and thereby affected function of HIF-1 signaling, which comprehensively altered risk and development of CRC. Moreover, smoking was also involved with CRC aggravation by modulating UCA1, miR-873 and HIF-1 signaling. Nonetheless, there were several flaws in the experimental design. Firstly, besides genotypes of rs12982687, the SW480 and SW620 cell lines utilized also differed in other genetic traits, so it might not be convincing enough to estimate the influence of rs12982687 genotype on CRC risk with the couple of cell lines. It was highly recommended that the identical CRC cell line with two genotypes of rs12982687 could be employed, which was unfortunately unfinished here due to technical deficiencies. Secondly, how rs12982687 affected miR-873’s regulation of UCA1 expression remained unclear, and if other mechanisms were involved in this modulation was still an uncertainty. Last but not the least, mice models were not established here to highlight the role of UCA1, miR-873 and HIF-1 signaling in CRC development. These deficiencies, after all, demanded further improvements.

## Supplementary information

**Additional file 1: Figure S1.** Multi-factor dimensionality reduction (MDR) model was established to assess the interactive effect of single nucleotide polymorphism rs12982687 (C > T) and environmental exposures on colorectal cancer (CRC) risk. (A) The rs12982687 could interact with smoking and alcohol to promote risk of CRC. The grayer the boxes, the more CRC risk. Within each box, bars on the left and on the right, respectively, represented case group and control group. The height of each bar was representative of sample size. (B) The hierarchical interaction graph was reflective of interaction degree among rs12982687, smoking and alcohol drinking. (C) The interaction dendrogram symbolized strength of synergy interaction.

**Additional file 2: Table S1.** Polymerase chain reaction amplification primer sequences.

**Additional file 3: Table S2.** Stratified analyses between rs12982687 and rs11085996 of UCA1 and susceptibility to colorectal cancer.

**Additional file 4: Table S3.** The optimal multifactor dimensionality reduction model for predicting onset of colorectal cancer.

**Additional file 5: Table S4.** KEGG pathways shared by genes targeted by miR-873-5p, miR-1207-5p and miR-584.

**Additional file 6:** Detailed results of stratified analyses regarding association of candidate SNPs in UCA1 with susceptibility to CRC and evaluating the interactive contribution of candidate SNPs in UCA1 and smoking to CRC risk by establishing GEI and MDR models.

## Data Availability

All data generated or analysed during this study are included in this published article and its supplementary information files.

## References

[1] Gilardi L, Vadrucci M (2017). Isolated Metachronous splenic metastasis from Colon Cancer found by 18F-FDG PET/CT. Clin Nucl Med.

[2] Naves T, Battu S, Jauberteau MO, Cardot PJ, Ratinaud MH, Verdier M (2012). Autophagic subpopulation sorting by sedimentation field-flow fractionation. Anal Chem.

[3] Kyo K, Maema A, Shirakawa M, Nakamura T, Koda K, Yokoyama H (2016). Pseudo-Meigs' syndrome secondary to metachronous ovarian metastases from transverse colon cancer. World J Gastroenterol.

[4] Negoi I, Runcanu A, Paun S, Beuran M (2016). Right Hemihepatectomy for Colon Cancer Metachronous liver metastasis in a patient with Crohn's disease: case report and review of the literature. Chirurgia.

[5] Schaukowitch K, Kim TK (2014). Emerging epigenetic mechanisms of long non-coding RNAs. Neuroscience.

[6] Kurihara M, Shiraishi A, Satake H, Kimura AP (2014). A conserved noncoding sequence can function as a spermatocyte-specific enhancer and a bidirectional promoter for a ubiquitously expressed gene and a testis-specific long noncoding RNA. J Mol Biol.

[7] Yang MH, Hu ZY, Xu C, Xie LY, Wang XY, Chen SY, Li ZG (1852). MALAT1 promotes colorectal cancer cell proliferation/migration/invasion via PRKA kinase anchor protein 9. Biochim Biophys Acta.

[8] Bernard D, Prasanth KV, Tripathi V, Colasse S, Nakamura T, Xuan Z, Zhang MQ, Sedel F, Jourdren L, Coulpier F (2010). A long nuclear-retained non-coding RNA regulates synaptogenesis by modulating gene expression. EMBO J.

[9] Tufarelli C, Stanley JA, Garrick D, Sharpe JA, Ayyub H, Wood WG, Higgs DR (2003). Transcription of antisense RNA leading to gene silencing and methylation as a novel cause of human genetic disease. Nat Genet.

[10] Tao K, Yang J, Hu Y, Sun Y, Tan Z, Duan J, Zhang F, Yan H, Deng A (2015). Clinical significance of urothelial carcinoma associated 1 in colon cancer. Int J Clin Exp Med.

[11] Jiang H, Wang Y, Ai M, Wang H, Duan Z, Wang H, Zhao L, Yu J, Ding Y, Wang S (2017). Long noncoding RNA CRNDE stabilized by hnRNPUL2 accelerates cell proliferation and migration in colorectal carcinoma via activating Ras/MAPK signaling pathways. Cell Death Dis.

[12] Ding J, Li J, Wang H, Tian Y, Xie M, He X, Ji H, Ma Z, Hui B, Wang K, Ji G (2017). Long noncoding RNA CRNDE promotes colorectal cancer cell proliferation via epigenetically silencing DUSP5/CDKN1A expression. Cell Death Dis.

[13] He X, Tan X, Wang X, Jin H, Liu L, Ma L, Yu H, Fan Z (2014). C-Myc-activated long noncoding RNA CCAT1 promotes colon cancer cell proliferation and invasion. Tumour Biol.

[14] Kasagi Y, Oki E, Ando K, Ito S, Iguchi T, Sugiyama M, Nakashima Y, Ohgaki K, Saeki H, Mimori K, Maehara Y (2017). The expression of CCAT2, a novel long noncoding RNA transcript, and rs6983267 single-nucleotide polymorphism genotypes in colorectal cancers. Oncology.

[15] Lu Y, Zhao X, Liu Q, Li C, Graves-Deal R, Cao Z, Singh B, Franklin JL, Wang J, Hu H (2017). lncRNA MIR100HG-derived miR-100 and miR-125b mediate cetuximab resistance via Wnt/beta-catenin signaling. Nat Med.

[16] Han Y, Yang YN, Yuan HH, Zhang TT, Sui H, Wei XL, Liu L, Huang P, Zhang WJ, Bai YX (2014). UCA1, a long non-coding RNA up-regulated in colorectal cancer influences cell proliferation, apoptosis and cell cycle distribution. Pathology.

[17] Yang X, Liu W, Xu X, Zhu J, Wu Y, Zhao K, He S, Li M, Wu Y, Zhang S (2018). Downregulation of long noncoding RNA UCA1 enhances the radiosensitivity and inhibits migration via suppression of epithelialmesenchymal transition in colorectal cancer cells. Oncol Rep.

[18] Nie W, Ge HJ, Yang XQ, Sun X, Huang H, Tao X, Chen WS, Li B (2016). LncRNA-UCA1 exerts oncogenic functions in non-small cell lung cancer by targeting miR-193a-3p. Cancer Lett.

[19] Fan Y, Shen B, Tan M, Mu X, Qin Y, Zhang F, Liu Y (2014). Long non-coding RNA UCA1 increases chemoresistance of bladder cancer cells by regulating Wnt signaling. FEBS J.

[20] Tuo YL, Li XM, Luo J (2015). Long noncoding RNA UCA1 modulates breast cancer cell growth and apoptosis through decreasing tumor suppressive miR-143. Eur Rev Med Pharmacol Sci.

[21] Fang Z, Wu L, Wang L, Yang Y, Meng Y, Yang H (2014). Increased expression of the long non-coding RNA UCA1 in tongue squamous cell carcinomas: a possible correlation with cancer metastasis. Oral Surg Oral Med Oral Pathol Oral Radiol.

[22] Jiao C, Song Z, Chen J, Zhong J, Cai W, Tian S, Chen S, Yi Y, Xiao Y (2016). lncRNA-UCA1 enhances cell proliferation through functioning as a ceRNA of Sox4 in esophageal cancer. Oncol Rep.

[23] Guo G, Kang Q, Zhu X, Chen Q, Wang X, Chen Y, Ouyang J, Zhang L, Tan H, Chen R (2015). A long noncoding RNA critically regulates Bcr-Abl-mediated cellular transformation by acting as a competitive endogenous RNA. Oncogene.

[24] Shuwen H, Qing Z, Yan Z, Xi Y (2018). Competitive endogenous RNA in colorectal cancer: a systematic review. Gene.

[25] Gong J, Liu W, Zhang J, Miao X, Guo AY (2015). lncRNASNP: a database of SNPs in lncRNAs and their potential functions in human and mouse. Nucleic Acids Res.

[26] Wu H, Zheng J, Deng J, Hu M, You Y, Li N, Li W, Lu J, Zhou Y (2013). A genetic polymorphism in lincRNA-uc003opf.1 is associated with susceptibility to esophageal squamous cell carcinoma in Chinese populations. Carcinogenesis.

[27] Samuelsson S, Finnstrom O, Flodmark O, Gaddlin PO, Leijon I, Wadsby M (2006). A longitudinal study of reading skills among very-low-birthweight children: is there a catch-up?. J Pediatr Psychol.

[28] Fried PA, Watkinson B, Siegel LS (1997). Reading and language in 9- to 12-year olds prenatally exposed to cigarettes and marijuana. Neurotoxicol Teratol.

[29] Zhang J, Schulz WA, Li Y, Wang R, Zotz R, Wen D, Siegel D, Ross D, Gabbert HE, Sarbia M (2003). Association of NAD(P)H: quinone oxidoreductase 1 (NQO1) C609T polymorphism with esophageal squamous cell carcinoma in a German Caucasian and a northern Chinese population. Carcinogenesis.

[30] Zhang J, Li Y, Wang R, Wen D, Sarbia M, Kuang G, Wu M, Wei L, He M, Zhang L, Wang S (2003). Association of cyclin D1 (G870A) polymorphism with susceptibility to esophageal and gastric cardiac carcinoma in a northern Chinese population. Int J Cancer.

[31] Song C, Xing D, Tan W, Wei Q, Lin D (2001). Methylenetetrahydrofolate reductase polymorphisms increase risk of esophageal squamous cell carcinoma in a Chinese population. Cancer Res.

[32] Miao X, Xing D, Tan W, Qi J, Lu W, Lin D (2002). Susceptibility to gastric cardia adenocarcinoma and genetic polymorphisms in methylenetetrahydrofolate reductase in an at-risk Chinese population. Cancer Epidemiol Biomark Prev.

[33] Tan W, Song N, Wang GQ, Liu Q, Tang HJ, Kadlubar FF, Lin DX (2000). Impact of genetic polymorphisms in cytochrome P450 2E1 and glutathione S-transferases M1, T1, and P1 on susceptibility to esophageal cancer among high-risk individuals in China. Cancer Epidemiol Biomark Prev.

[34] Khoury MJ, Wagener DK (1995). Epidemiological evaluation of the use of genetics to improve the predictive value of disease risk factors. Am J Hum Genet.

[35] Ottman Ruth, Rao D. C. (1990). An epidemiologic approach to gene-environment interaction. Genetic Epidemiology.

[36] Hahn LW, Ritchie MD, Moore JH (2003). Multifactor dimensionality reduction software for detecting gene-gene and gene-environment interactions. Bioinformatics.

[37] Zhang D, Lei J, Ma J, Chen X, Sheng L, Jiang Z, Nan L, Xu Q, Duan W, Wang Z (2016). Beta2-adrenogenic signaling regulates NNK-induced pancreatic cancer progression via upregulation of HIF-1alpha. Oncotarget.

[38] Gortz GE, Horstmann M, Aniol B, Reyes BD, Fandrey J, Eckstein A, Berchner-Pfannschmidt U (2016). Hypoxia-dependent HIF-1 activation impacts on tissue remodeling in Graves' Ophthalmopathy-implications for smoking. J Clin Endocrinol Metab.

[39] Volm M, Koomagi R (2000). Hypoxia-inducible factor (HIF-1) and its relationship to apoptosis and proliferation in lung cancer. Anticancer Res.

[40] Chen H, Feng J, Zhang Y, Shen A, Chen Y, Lin J, Lin W, Sferra TJ, Peng J (2015). Pien Tze Huang inhibits hypoxia-induced angiogenesis via HIF-1 alpha /VEGF-A pathway in colorectal Cancer. Evid Based Complement Alternat Med.

[41] Li T, Xiao Y, Huang T (2018). HIF1alphainduced upregulation of lncRNA UCA1 promotes cell growth in osteosarcoma by inactivating the PTEN/AKT signaling pathway. Oncol Rep.

[42] Li X, Wu Y, Liu A, Tang X (2016). Long non-coding RNA UCA1 enhances tamoxifen resistance in breast cancer cells through a miR-18a-HIF1alpha feedback regulatory loop. Tumour Biol.

[43] Wang F, Ying HQ, He BS, Pan YQ, Deng QW, Sun HL, Chen J, Liu X, Wang SK (2015). Upregulated lncRNA-UCA1 contributes to progression of hepatocellular carcinoma through inhibition of miR-216b and activation of FGFR1/ERK signaling pathway. Oncotarget.

[44] Wang F, Zhou J, Xie X, Hu J, Chen L, Hu Q, Guo H, Yu C (2015). Involvement of SRPK1 in cisplatin resistance related to long non-coding RNA UCA1 in human ovarian cancer cells. Neoplasma.

[45] Huang J, Zhou N, Watabe K, Lu Z, Wu F, Xu M, Mo YY (2014). Long non-coding RNA UCA1 promotes breast tumor growth by suppression of p27 (Kip1). Cell Death Dis.

[46] Zheng Q, Wu F, Dai WY, Zheng DC, Zheng C, Ye H, Zhou B, Chen JJ, Chen P (2015). Aberrant expression of UCA1 in gastric cancer and its clinical significance. Clin Transl Oncol.

[47] Li JY, Ma X, Zhang CB (2014). Overexpression of long non-coding RNA UCA1 predicts a poor prognosis in patients with esophageal squamous cell carcinoma. Int J Clin Exp Pathol.

[48] Tian Y, Zhang X, Hao Y, Fang Z, He Y (2014). Potential roles of abnormally expressed long noncoding RNA UCA1 and Malat-1 in metastasis of melanoma. Melanoma Res.

[49] Wang XS, Zhang Z, Wang HC, Cai JL, Xu QW, Li MQ, Chen YC, Qian XP, Lu TJ, Yu LZ (2006). Rapid identification of UCA1 as a very sensitive and specific unique marker for human bladder carcinoma. Clin Cancer Res.

[50] Wang F, Li X, Xie X, Zhao L, Chen W (2008). UCA1, a non-protein-coding RNA up-regulated in bladder carcinoma and embryo, influencing cell growth and promoting invasion. FEBS Lett.

[51] Pillai RS, Bhattacharyya SN, Artus CG, Zoller T, Cougot N, Basyuk E, Bertrand E, Filipowicz W (2005). Inhibition of translational initiation by Let-7 MicroRNA in human cells. Science.

[52] Zhang EB, Kong R, Yin DD, You LH, Sun M, Han L, Xu TP, Xia R, Yang JS, De W, Chen J (2014). Long noncoding RNA ANRIL indicates a poor prognosis of gastric cancer and promotes tumor growth by epigenetically silencing of miR-99a/miR-449a. Oncotarget.

[53] Wang J, Liu X, Wu H, Ni P, Gu Z, Qiao Y, Chen N, Sun F, Fan Q (2010). CREB up-regulates long non-coding RNA, HULC expression through interaction with microRNA-372 in liver cancer. Nucleic Acids Res.

[54] Luo J, Zhu H, Jiang H, Cui Y, Wang M, Ni X, Ma C (2018). The effects of aberrant expression of LncRNA DGCR5/miR-873-5p/TUSC3 in lung cancer cell progression. Cancer Med.

[55] Cao D, Yu T, Ou X (2016). MiR-873-5P controls gastric cancer progression by targeting hedgehog-GLI signaling. Pharmazie.

[56] Hirota K, Semenza GL (2006). Regulation of angiogenesis by hypoxia-inducible factor 1. Crit Rev Oncol Hematol.

[57] Semenza GL (2010). HIF-1: upstream and downstream of cancer metabolism. Curr Opin Genet Dev.

[58] Wei D, Peng JJ, Gao H, Li H, Li D, Tan Y, Zhang T (2013). Digoxin downregulates NDRG1 and VEGF through the inhibition of HIF-1alpha under hypoxic conditions in human lung adenocarcinoma A549 cells. Int J Mol Sci.

[59] Metcalfe RA, Weetman AP (1994). Stimulation of extraocular muscle fibroblasts by cytokines and hypoxia: possible role in thyroid-associated ophthalmopathy. Clin Endocrinol.

[60] Chng CL, Chew CS, Peh YP, Fook-Chong SM, Seah LL, Khoo DH, Lai OF (2014). Hypoxia increases adipogenesis and affects adipocytokine production in orbital fibroblasts-a possible explanation of the link between smoking and Graves' ophthalmopathy. Int J Ophthalmol.

[61] Shannon AM, Bouchier-Hayes DJ, Condron CM, Toomey D (2003). Tumour hypoxia, chemotherapeutic resistance and hypoxia-related therapies. Cancer Treat Rev.

[62] Goethals L, Debucquoy A, Perneel C, Geboes K, Ectors N, De Schutter H, Penninckx F, McBride WH, Begg AC, Haustermans KM (2006). Hypoxia in human colorectal adenocarcinoma: comparison between extrinsic and potential intrinsic hypoxia markers. Int J Radiat Oncol Biol Phys.

[63] Mizukami Y, Fujiki K, Duerr EM, Gala M, Jo WS, Zhang X, Chung DC (2006). Hypoxic regulation of vascular endothelial growth factor through the induction of phosphatidylinositol 3-kinase/rho/ROCK and c-Myc. J Biol Chem.

[64] Abramovitch R, Tavor E, Jacob-Hirsch J, Zeira E, Amariglio N, Pappo O, Rechavi G, Galun E, Honigman A (2004). A pivotal role of cyclic AMP-responsive element binding protein in tumor progression. Cancer Res.

[65] Caro J (2001). Hypoxia regulation of gene transcription. High Alt Med Biol.

[66] Roberts DL, Williams KJ, Cowen RL, Barathova M, Eustace AJ, Brittain-Dissont S, Tilby MJ, Pearson DG, Ottley CJ, Stratford IJ, Dive C (2009). Contribution of HIF-1 and drug penetrance to oxaliplatin resistance in hypoxic colorectal cancer cells. Br J Cancer.

[67] Wincewicz A, Sulkowska M, Koda M, Sulkowski S (2007). Cumulative expression of HIF-1-alpha, Bax, Bcl-xL and P53 in human colorectal cancer. Pathology.

[68] Zhang W, Shi X, Peng Y, Wu M, Zhang P, Xie R, Wu Y, Yan Q, Liu S, Wang J (2015). HIF-1alpha promotes epithelial-Mesenchymal transition and metastasis through direct regulation of ZEB1 in colorectal Cancer. PLoS One.

