# Supplementary figures and images for: SNP rs12982687 affects binding capacity of lncRNA UCA1 with miR-873-5p: involvement in smoking-triggered colorectal cancer progression
Source: Cell Commun Signal. 2020 Mar 6;18:37. doi: 10.1186/s12964-020-0518-0 (PMC7059387; doi:10.1186/s12964-020-0518-0)

**(A)**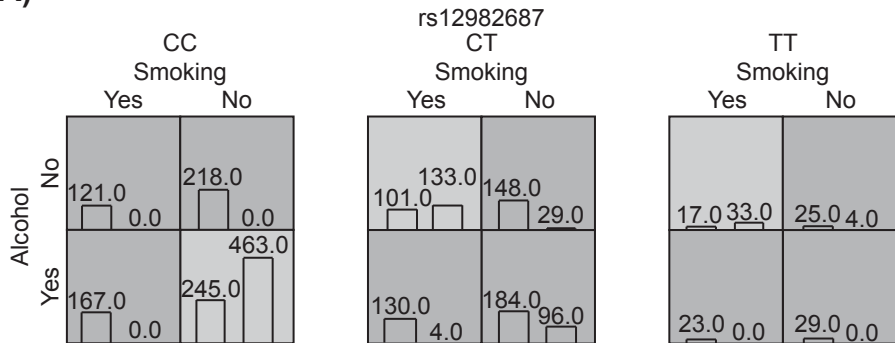**(B)**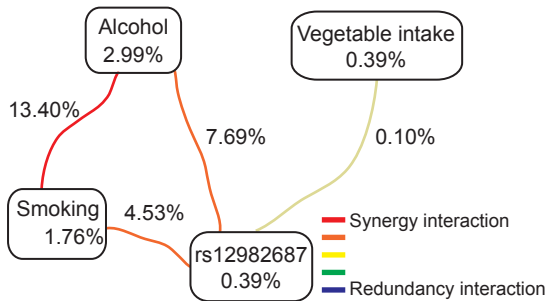**(C)**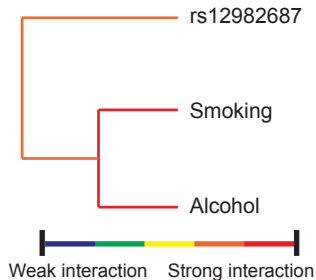

Supplement: Supplementary file 1 — Additional file 1: Figure S1. Multi-factor dimensionality reduction (MDR) model was established to assess the interactive effect of single nucleotide polymorphism rs12982687 (C > T) and environmental exposures on colorectal cancer (CRC) risk. (A) The rs12982687 could interact with smoking and alcohol to promote risk of CRC. The grayer the boxes, the more CRC risk. Within each box, bars on the left and on the right, respectively, represented case group and control group. The height of each bar was representative of sample size. (B) The hierarchical interaction graph was reflective of interaction degree among rs12982687, smoking and alcohol drinking. (C) The interaction dendrogram symbolized strength of synergy interaction. [file 12964_2020_518_MOESM1_ESM.pdf]
