# Supplementary material for: SNP rs12982687 affects binding capacity of lncRNA UCA1 with miR-873-5p: involvement in smoking-triggered colorectal cancer progression
Source: Cell Commun Signal. 2020 Mar 6;18:37. doi: 10.1186/s12964-020-0518-0 (PMC7059387; doi:10.1186/s12964-020-0518-0)
Supplement: Supplementary file 2 — Additional file 2: Table S1. Polymerase chain reaction amplification primer sequences. [file 12964_2020_518_MOESM2_ESM.docx]

**Supplementary Table 1 Polymerase chain reaction amplification primer sequences**

| **Gene** | **Primer** |
| --- | --- |
| UCA1 | Forward: 5'-CTCTCCATTGGGTTCACCATTC-3' |
|  | Reverse: 5'-GCGGCAGGTCTTAAGAGATGAG-3' |
| HIF-1α | Forward: 5'-GCTTACACACAGAAATGGCC -3' |
|  | Reverse: 5'-AGCACCTTCCACGTTGCTGA -3' |
| GAPDH | Forward: 5'-TCGACAGTCAGCCGCATCTTCTTT-3' |
|  | Reverse: 5'-ACCAAATCCGTTGACTCCGACCTT-3' |
| miR-873-5p | Forward: 5'-ACACTCCAGCTGGGGCAGGAACTTGTGAG-3' |
|  | Reverse: 5'-TGGTGTCGTGGAGTCG-3' |
| miR-1207-5p | Forward: 5'-TCCGAAGGCAGGGAGGCAG-3' |
|  | Reverse: 5'-GTGCAGGGTCCGAGGT-3' |
| miR-584 | Forward: 5′-TGCAATGTGTGTGTTAGCCA-3′ |
|  | Reverse: 5′-ATCATTGCTCCTTGGCTGGT-3′ |
| U6 | Forward: 5'-CTCGCTTCGGCAGCACA-3' |
|  | Reverse: 5'-AACGCTTCACGAATTTGCGT-3' |
