# Supplementary material for: SNP rs12982687 affects binding capacity of lncRNA UCA1 with miR-873-5p: involvement in smoking-triggered colorectal cancer progression
Source: Cell Commun Signal. 2020 Mar 6;18:37. doi: 10.1186/s12964-020-0518-0 (PMC7059387; doi:10.1186/s12964-020-0518-0)
Supplement: Supplementary file 3 — Additional file 3: Table S2. Stratified analyses between rs12982687 and rs11085996 of UCA1 and susceptibility to colorectal cancer. [file 12964_2020_518_MOESM3_ESM.docx]

**Supplementary Table 2 Stratified analyses between rs12982687 and rs11085996 of UCA1 and susceptibility to colorectal cancer.**

| **Clinical feature** | **Classification** | **rs12982687** | | | | | **rs11085996** | | | | |
| --- | --- | --- | --- | --- | --- | --- | --- | --- | --- | --- | --- |
|  |  | **CRC genotype** | | **Control genotype** | | **OR (95% CI)*** | **CRC genotype** | | **Control genotype** | | **OR (95% CI)*** |
|  |  | **CC** | **CT+TT** | **CC** | **CT+TT** |  | **AA** | **AG+GG** | **AA** | **AG+GG** |  |
| Age (years) | ≥50 | 198 | 225 | 243 | 492 | **0.64 (0.48-0.85)** ^a^ | 281 | 142 | 448 | 287 | 0.76 (0.57-1.02) ^a^ |
|  | ＜50 | 265 | 74 | 508 | 165 | 1.12 (0.79-1.58) ^a^ | 240 | 99 | 497 | 176 | 1.02 (0.74-1.39) ^a^ |
| Gender | Female | 111 | 78 | 151 | 150 | 0.88 (0.56-1.37) ^b^ | 101 | 88 | 175 | 126 | 1.08 (0.72-1.63) ^b^ |
|  | Male | 352 | 221 | 600 | 507 | **0.75 (0.58-0.97)** ^b^ | 420 | 153 | 770 | 337 | 0.81 (0.64-1.03) ^b^ |
| Smoking | Yes | 339 | 253 | 383 | 466 | **0.70 (0.54-0.90)** ^c^ | 399 | 193 | 606 | 243 | 1.09 (0.86-1.39) ^c^ |
|  | No | 124 | 46 | 368 | 191 | 1.03 (0.66-1.60) ^c^ | 122 | 48 | 339 | 220 | **0.54 (0.35-0.82)** ^c^ |
| Alcohol | Yes | 367 | 196 | 457 | 321 | **0.69 (0.53-0.91)** ^d^ | 389 | 174 | 528 | 250 | 0.87 (0.68-1.12) ^d^ |
|  | No | 96 | 103 | 294 | 336 | 1.10 (0.74-1.64) ^d^ | 132 | 67 | 417 | 213 | 1.08 (0.74-1.58) ^d^ |
| Fruit intake, >1 serving/day | Yes | 158 | 171 | 345 | 476 | 0.80 (0.59-1.08) ^e^ | 199 | 130 | 620 | 201 | **0.67 (0.52-0.87)** ^e^ |
|  | No | 305 | 128 | 406 | 181 | 0.76 (0.55-1.05) ^e^ | 322 | 111 | 325 | 262 | 1.27 (0.96-1.67) ^e^ |
| Vegetable intake, >1 serving/day | Yes | 262 | 219 | 515 | 475 | 0.83 (0.64-1.09) ^f^ | 311 | 170 | 622 | 368 | **0.75 (0.58-0.97)** ^f^ |
|  | No | 201 | 80 | 236 | 182 | 0.70 (0.47-1.05) ^f^ | 210 | 71 | 323 | 95 | 0.97 (0.66-1.43) ^f^ |
| Foods or beverages temperature | Hot | 344 | 150 | 498 | 286 | **0.77 (0.58-1.02)** ^g^ | 336 | 158 | 483 | 301 | **0.75 (0.58-0.98)** ^g^ |
|  | Warm | 119 | 149 | 253 | 371 | 0.80 (0.57-1.13) ^g^ | 185 | 83 | 462 | 162 | 1.15 (0.82-1.61) ^g^ |
| Smoked or pickled foods intake | Yes | 319 | 159 | 486 | 348 | **0.67 (0.51-0.89)** ^h^ | 300 | 178 | 500 | 334 | 0.82 (0.63-1.05) ^h^ |
|  | No | 144 | 140 | 265 | 309 | 1.02 (0.71-1.47) ^h^ | 221 | 63 | 445 | 129 | 1.02 (0.71-1.47) ^h^ |

* OR=(Heterozygote+Mutant homozygote)/Wild homozygote; ^a^ Gender, smoking, alcohol, fruit intake, vegetable intake, food/beverage temperature and smoked food adjusted OR; ^b^ Age, smoking, alcohol, fruit intake, vegetable intake, food/beverage temperature and smoked food adjusted OR; ^c^ Age, gender, alcohol, fruit intake, vegetable intake, food/beverage temperature and smoked food adjusted OR; ^d^ Age, gender, smoking, fruit intake, vegetable intake, food/beverage temperature and smoked food adjusted OR; ^e^ Age, gender, smoking, alcohol, vegetable intake, food/beverage temperature and smoked food adjusted OR; ^f^ Age, gender, smoking, alcohol, fruit intake, food/beverage temperature and smoked food adjusted OR; ^g^ Age, gender, smoking, alcohol, fruit intake, vegetable intake and smoked food adjusted OR; ^h^ Age, gender, smoking, alcohol, fruit intake, vegetable intake and food/beverage temperature adjusted OR.
