# Supplementary material for: SNP rs12982687 affects binding capacity of lncRNA UCA1 with miR-873-5p: involvement in smoking-triggered colorectal cancer progression
Source: Cell Commun Signal. 2020 Mar 6;18:37. doi: 10.1186/s12964-020-0518-0 (PMC7059387; doi:10.1186/s12964-020-0518-0)
Supplement: Supplementary file 4 — Additional file 4: Table S3. The optimal multifactor dimensionality reduction model for predicting onset of colorectal cancer. [file 12964_2020_518_MOESM4_ESM.docx]

**Supplementary Table 3 The optimal multifactor dimensionality reduction model for predicting onset of colorectal cancer.**

| **Best model** | **Training accuracy (%)** | **Testing accuracy (%)** | **CVC** | **χ^2^** | ***p* value** | **OR** | **95% CI** |
| --- | --- | --- | --- | --- | --- | --- | --- |
| Alcohol | 59.31 | 59.31 | 10/10 | 1.62 | 0.203 | **1.48** | **0.81-2.73** |
| Smoking, Alcohol | 72.82 | 72.82 | 10/10 | 37.97 | <0.001 | **15.39** | **5.33-44.43** |
| Smoking, Alcohol, rs12982687 | 78.42 | 77.58 | 10/10 | 44.04 | <0.001 | **7.91** | **4.14-15.12** |

CVC: Cross-validation consistency; OR: Odds ratio; CI: confidence interval.
