# Supplementary material for: SNP rs12982687 affects binding capacity of lncRNA UCA1 with miR-873-5p: involvement in smoking-triggered colorectal cancer progression
Source: Cell Commun Signal. 2020 Mar 6;18:37. doi: 10.1186/s12964-020-0518-0 (PMC7059387; doi:10.1186/s12964-020-0518-0)
Supplement: Supplementary file 5 — Additional file 5: Table S4. KEGG pathways shared by genes targeted by miR-873-5p, miR-1207-5p and miR-584. [file 12964_2020_518_MOESM5_ESM.docx]

**Supplementary Table 4 KEGG pathways shared by genes targeted by miR-873-5p, miR-1207-5p and miR-584**

| **KEGG pathway** | ***P* value** | **Genes** |
| --- | --- | --- |
| Prion Diseases (hsa05020) | <0.001 | 3 |
| Lysine Degradation (hsa00310) | <0.001 | 11 |
| Steroid Biosynthesis (hsa00100) | <0.001 | 3 |
| Glycosaminoglycan Biosynthesis-Keratan Sulfate (hsa00533) | 0.001 | 3 |
| Adherens Junction (hsa04520) | 0.001 | 16 |
| Arrhythmogenic right ventricular cardiomyopathy (ARVC) (hsa05412) | 0.004 | 8 |
| Proteoglycans in cancer (hsa05205) | 0.004 | 18 |
| Gap junction (hsa04540) | 0.009 | 10 |
| HIF-1 signaling pathway (hsa04066) | 0.009 | 18 |
| Glioma (hsa05214) | 0.009 | 11 |
| Estrogen signaling pathway (hsa04915) | 0.018 | 13 |
| Viral carcinogenesis (hsa05203) | 0.037 | 24 |
| Prostate Cancer (hsa05215) | 0.046 | 15 |
