# Supplementary material for: SNP rs12982687 affects binding capacity of lncRNA UCA1 with miR-873-5p: involvement in smoking-triggered colorectal cancer progression
Source: Cell Commun Signal. 2020 Mar 6;18:37. doi: 10.1186/s12964-020-0518-0 (PMC7059387; doi:10.1186/s12964-020-0518-0)
Supplement: Supplementary file 6 — Additional file 6: Detailed results of stratified analyses regarding association of candidate SNPs in UCA1 with susceptibility to CRC and evaluating the interactive contribution of candidate SNPs in UCA1 and smoking to CRC risk by establishing GEI and MDR models. [file 12964_2020_518_MOESM6_ESM.docx]

**Stratified analyses regarding association of candidate SNPs in UCA1 with susceptibility to CRC**

Among the population aged ≥ 50 years old, we found that genotypes TT and CT of rs12982687 could significantly lower CRC risk (OR=0.64, 95% CI: 0.48-0.85, *P*<0.05) (Supplementary Table 2). Similarly, genotypes TT and CT served as a protective parameter against CRC susceptibility among the population who possessed habits of smoking (OR=0.70, 95% CI: 0.54-0.90), alcohol consumption (OR=0.69, 95% CI: 0.53-0.91) and intake of smoked or pickled food (OR=0.67, 95% CI: 0.51-0.89). Besides, among CRC subjects characterized by non-smoking (OR=0.54, 95% CI: 0.35-0.82), consumption of fruit/vegetable (OR=0.67, 95% CI: 0.52-0.87; OR=0.75, 95% CI: 0.58-0.97) and intake of hot food (OR=0.75, 95% CI: 0.58-0.98), genotypes AG and GG of rs11085996 were associated with decreased risk of CRC, when compared with homozygote AA (Supplementary Table 2).

**Interaction of candidate SNPs in UCA1 and smoking on susceptibility to CRC based on MDR model**

As was demonstrated in Supplementary Table 3 and Supplementary Figure 1A, the MDR model was devised to evaluate the comprehensive impacts of rs12982687 and habitual parameters (i.e. gender, smoking, alcohol consumption, fruit intake, vegetable intake and temperature of food or beverage) on CRC risk. It turned out that rs12982687, smoking and alcohol consumption constituted the optimum interactive model with a testing accuracy of 77.58% and a cross-consistency of 10/10. Besides, rs12982687 (CC) and smoking (Yes) or alcohol (Yes) seemed as the strongest combination in inducing CRC risk (Supplementary Figure 1B-C).
